# Compromised cardiopulmonary resuscitation quality due to regurgitation during endotracheal intubation: a randomised crossover manikin simulation study

**DOI:** 10.1186/s12873-022-00662-0

**Published:** 2022-07-09

**Authors:** Li-Wei Lin, James DuCanto, Chen-Yang Hsu, Yung-Cheng Su, Chi-Chieh Huang, Shih-Wen Hung

**Affiliations:** 1 Emergency Department, Su Memorial Hospital, Shin-Kong Wu Ho, Taipei, Taiwan; 2 School of Medicine, College of Medicine, Fu Jen Catholic University, New Taipei, Taiwan; 3 CrazyatLAB (Critical Airway Training Laboratory), Taipei, Taiwan; 4 Advocate Aurora Medical Group, Milwaukee, WI USA; 5 Dachung Hospital, Miaoli, Taiwan; 6 Master of Public Health Program, National Taiwan University, Taipei, Taiwan; 7 School of Medicine, Tzu Chi University, Hualien, Taiwan; 8 Emergency Department, Dalin Tzu Chi Hospital, Buddhist Tzu Chi Medical Foundation, Chiayi, Taiwan

**Keywords:** Airway management, Cardiopulmonary resuscitation, Manikin, Regurgitation

## Abstract

**Background:**

Regurgitation is a complication common during cardiopulmonary resuscitation (CPR). This manikin study evaluated the effect of regurgitation during endotracheal intubation on CPR quality.

**Methods:**

An airway-CPR manikin was modified to regurgitate simulated gastric contents into the oropharynx during chest compression during CPR. In total, 54 emergency medical technician-paramedics were assigned to either an oropharyngeal regurgitation or clean airway scenario and then switched to the other scenario after finishing the first. The primary outcomes were CPR quality metrics, including chest compression fraction (CCF), chest compression depth, chest compression rate, and longest interruption time. The secondary outcomes were intubation success rate and intubation time.

**Results:**

During the first CPR–intubation sequence, the oropharyngeal regurgitation scenario was associated with a significantly lower CCF (79.6% vs. 85.1%, *P* < 0.001), compression depth (5.2 vs. 5.4 cm, *P* < 0.001), and first-pass success rate (35.2% vs. 79.6%, *P* < 0.001) and greater longest interruption duration (4.0 vs. 3.0 s, *P* < 0.001) than the clean airway scenario. During the second and third sequences, no significant difference was observed in the CPR quality metrics between the two scenarios. In the oropharyngeal regurgitation scenario, successful intubation was independently and significantly associated with compression depth (hazard ratio = 0.47, 95% confidence interval, 0.24–0.91), whereas none of the CPR quality metrics were related to successful intubation in the clean airway scenario.

**Conclusion:**

Regurgitation during endotracheal intubation significantly reduces CPR quality.

**Trial registration:**

ClinicalTrials.gov, NCT05278923, March 14, 2022.

**Supplementary Information:**

The online version contains supplementary material available at 10.1186/s12873-022-00662-0.

## Introduction

Regurgitation is an adverse event common during cardiopulmonary resuscitation (CPR) and occurs in 20%–32% of patients experiencing out-of-hospital cardiac arrest (OHCA) [1–4]. Regurgitation during CPR may result from gastric insufflation from mouth-to-mouth or bag valve mask (BVM) ventilation, loss of lower oesophageal sphincter tone after cardiac arrest, or intra-abdominal pressure increased by chest compression [3]. Regurgitation can impair ventilation, induce aspiration, and decrease survival to hospital discharge [2]. Gastric fluid in the airway obscures the laryngeal view, thereby considerably decreasing the first-pass success of endotracheal intubation (ETI) by paramedics [5].

A human cadaver study reported that ETI outperforms other airway management devices, such as the i-gel, laryngeal mask, and laryngeal tube, in preventing aspiration when regurgitation occurs during CPR [6]. However, ETI is also associated with multiple and prolonged CPR pauses [7]. Compared with the use of supraglottic airway (SGA) devices, ETI results in more hands-off time during CPR [8, 9]. Recent randomised clinical trials have revealed that airway management with an SGA device provides superior outcomes to those of ETI in patients with OHCA [10]. However, ETI remains the preferred management strategy for an airway affected by regurgitation in patients with OHCA [11].

Current guidelines focus on the quality of CPR because it is a key determinant of survival in patients with OHCA [12, 13]. However, evidence regarding the impact of regurgitation during ETI on CPR quality is limited. This manikin simulation study assessed CPR quality during ETI in airways with and without regurgitation.

## Materials and methods

### Participants

This randomised crossover manikin simulation study was approved the Institutional Review Board of Shin Kong Wu Ho-Su Memorial Hospital (No. 20180503R) and registered in ClinicalTrials.gov (NCT05278923, first submitted date: March 14, 2022). The participants were emergency medical technician-paramedics (EMT-Ps) experienced in advanced airway management and CPR, working for the emergency medical services in New Taipei City, Taiwan, which make more than 135,000 dispatches every year. This study was performed from October 2018 to May 2019, and all participants provided written informed consent.

### Patient and public involvement

Patients or the public were not involved in the design, or conduct, or reporting, or dissemination plans of our research.

### Simulation setup

**Fig. 1:**
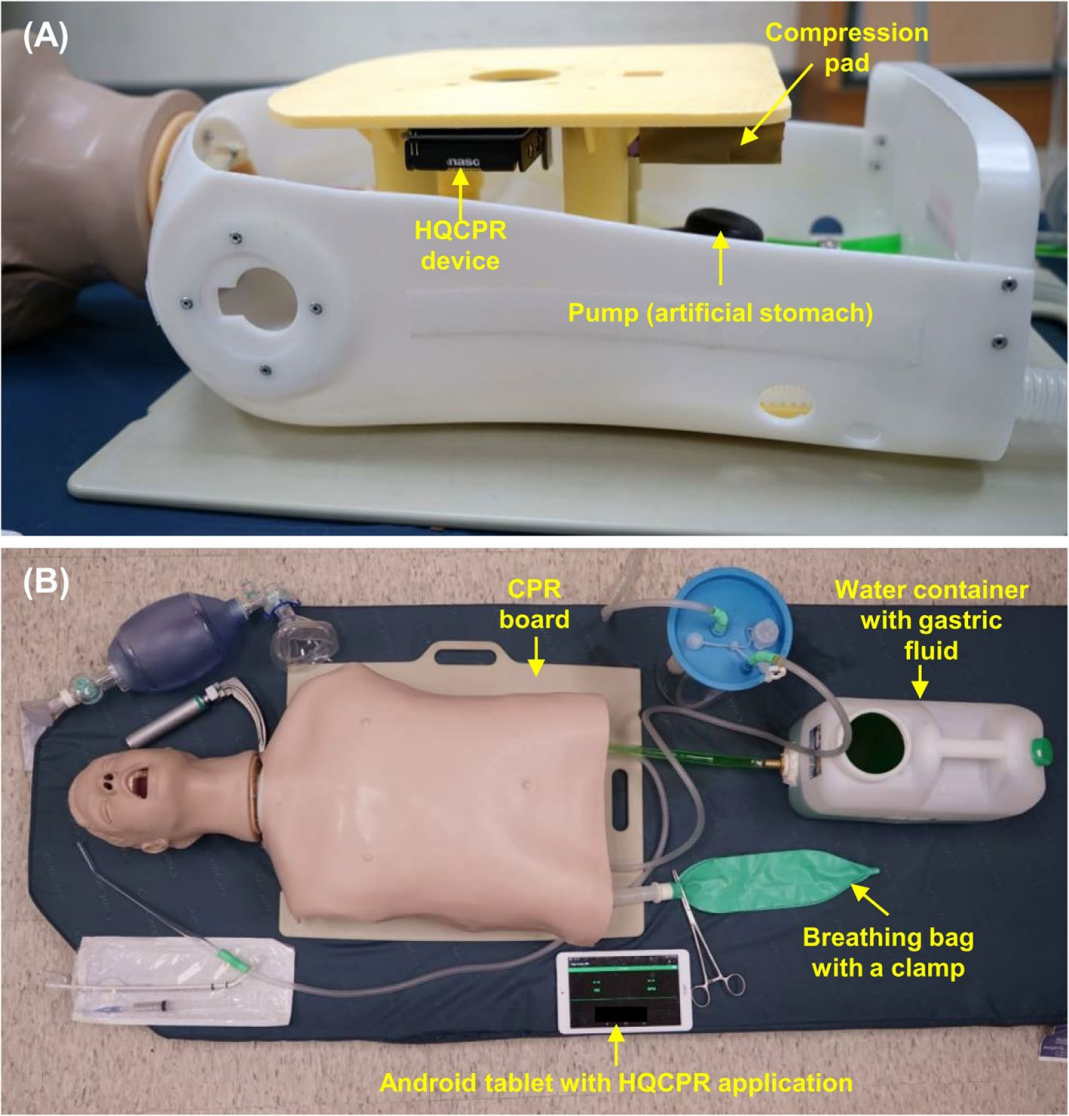
Manikin setup: **A** The outflow port of the pump (artificial stomach) was connected to the oesophagus, and the inflow port of the pump was connected to the water container via vinyl tubes. The right main bronchus was connected to an anaesthesia breathing bag through a breathing circuit, and the left main bronchus was occluded. The HQCPR device was attached to the inside of the compression plate. The compression pad was attached to the bottom of the compression plate for squeezing the pump during chest compression. **B** During the simulations, the HQCPR application on the Android tablet recorded the CPR quality metrics. The anaesthesia breathing bag was unclamped only for checking intubation. Abbreviations: CPR, cardiopulmonary resuscitation

An airway-CPR manikin (Airway Larry Airway Management Trainer Torso, Nasco, Fort Atkinson, WI, USA) was modified to simulate oropharyngeal regurgitation during CPR (Fig. 1). A manual pump was fixed on the bottom of the torso to simulate the stomach. A clear vinyl tube (inner diameter: 12 mm, outer diameter: 17 mm) was connected the manikin’s oesophagus and the outflow port of the pump. A water container outside the manikin was filled with simulated gastric content (3 g of xanthan gum per litre of water, plus green food colouring) and connected to the inflow port of the pump through another vinyl tube. The manikin’s left main bronchus was occluded using a red cap provided by the manufacturer. The lung was simulated by an anaesthesia breathing bag placed outside the manikin and connected to the manikin’s right main bronchus via a breathing circuit. A compression pad was attached to the bottom of the manikin’s compression plate. During chest compression, the compression pad squeezed the pump to regurgitate gastric contents into the oropharynx (Supplementary Video 1). The compression pad thickness ensured a compression depth more than 6 cm and allowed complete chest recoil. To avoid continuous drainage of the regurgitated gastric fluid from the oropharynx into the lung, the simulated lung was closed with a surgical clamp and opened only to check the position of the endotracheal tube by squeezing the BVM. Intubation was performed only after the first five cycles of CPR to ensure the simulated gastric fluid reached the oropharyngeal cavity during chest compression. For simulating head movement during chest compression, the manikin was laid on a ward bed mattress, and a CPR board was placed under its torso. During chest compression, the elastic mattress induced head movement, and the CPR board diminished the compression force absorbed by the mattress. An HQCPR device (Taiwan Paramedicine Service Co., Ltd., Taoyuan, Taiwan), comprising an optical distance sensor, Bluetooth module, and battery box, was attached to the inside of the chest compression plate. During chest compression, the device could detect the distance between the compression plate and the bottom of the manikin torso and transmit the signals to an Android tablet (Android version 4.4–5.0) via Bluetooth. The HQCPR application on the Android device recorded the rate, the depth, and any interruption of chest compression. For performing ETI, a direct laryngoscope with a standard Macintosh 4 blade was used. A 7.5-mm cuffed endotracheal tube, lubricated with gel lubricant, and a stylet were used for intubation. In the oropharyngeal regurgitation scenario (see the next section for details), a Yankauer catheter connected to a wall-mounted suction device was available to suction the regurgitated gastric fluid from the airway.

### Study protocol and simulation

Two scenarios were simulated. CPR and ETI in an airway with regurgitation (oropharyngeal regurgitation scenario) and CPR and ETI in an airway without regurgitation (clean airway scenario). All EMT-Ps were randomly assigned to participate in one of the scenarios. Randomization was performed using simple randomization method via a random number table. After all EMT-Ps had finished the first scenario, they switched to the other. Three EMT-Ps formed a resuscitation team and played one of the following roles: airway manager, first compressor, or second compressor. In each scenario, each EMT-P was required to take a turn playing all three roles. During each simulation, the airway manager performed BVM ventilation, and the first and second compressors alternately provided chest compression for every five cycles of CPR, with a compression-to-ventilation ratio of 30:2 (Supplementary Fig. 1).

After the first five cycles of CPR, the airway manager was asked to perform intubation during the ongoing chest compression to minimise intubation-associated interruption of chest compression. If necessary, the airway manager could request a pause of the ongoing chest compression. If intubation was not successful, the airway manager performed BVM ventilation twice and then reattempt intubation. After intubation, the airway manager used the BVM to check the lung distention to confirm successful intubation. Each simulation was ended after successful or failed intubation. Failed intubation was defined as either oesophageal intubation or three unsuccessful attempts.

### Measurement

The primary outcomes were CPR quality metrics, namely chest compression fraction (CCF), chest compression depth, chest compression rate, and longest interruption time. The secondary outcomes were the intubation success rate and intubation time.

Each CPR–intubation sequence comprised two segments: a compression segment and a hands-off segment. Supplementary Fig. 2 presents the rules for determining the start and end of a CPR–intubation sequence. CCF was defined as the proportion of time spent on chest compression in each CPR–intubation sequence. The longest interruption time was defined as the longest hands-off duration in each sequence. An intubation attempt was defined as the insertion of the laryngoscope blade into the mouth and its subsequent withdrawal from the mouth. Intubation time was defined as the period between the start and the end of an intubation attempt. The time spent checking the endotracheal tube position by manual ventilation through the endotracheal tube was not included in the CPR–intubation sequence.

Two video cameras were setup to record the entire simulation process. Two observers reviewed the video records independently to identify the start and end of each CPR–intubation sequence, any intubation attempts, and the hands-off and compression segments of each sequence. Disagreements were resolved by reaching mutual consensus. The HQCPR application on an Android device recorded chest compression depth, rate, and interruptions (defined as no chest compression [hands off] for > 1 s). The data from both the video recording and the HQCPR application were used in subsequent analysis.

### Statistical analysis

Continuous data are presented as medians with interquartile ranges, and categorical data are presented as frequency counts and percentages. The continuous data were compared using the Wilcoxon rank sum test in the first CPR–intubation sequence and the Mann–Whitney U test in the second and third sequences. The McNemar test was used to compare the intubation success rate in the first sequence, and Pearson’s chi-square test was used for the second and third sequences. The continuous data from all three CPR–intubation sequences were compared using the Kruskal–Wallis test. Post hoc analysis was performed using Conover’s test.

The association of CPR quality metrics with successful intubation was evaluated using a Cox proportional hazards regression model. The results of multivariate analyses are presented as hazard ratios (HRs) with corresponding 95% confidence intervals (CIs). A two-tailed *P* < 0.05 indicates statistical significance. MedCalc Statistical Software version 19.2. (MedCalc Software, Ostend, Belgium) was used for data analysis. All statistical analyses were performed by one of the authors (Y.C. Su).

## Results

In total, 54 EMT-Ps participated in this study, with experience ranging from 2 to 17 years (median 10 years). In the first CPR–intubation sequence, the oropharyngeal regurgitation scenario had a significantly lower CCF (79.6% vs. 85.1%, *P* < 0.001), compression depth (5.2 vs. 5.4 cm, *P* < 0.001), and first-pass intubation success rate (35.2% vs. 79.6%, *P* < 0.001) and greater longest interruption duration (4.0 vs. 3.0 s, *P* < 0.001) than the clean airway scenario. The compression rate and intubation time did not significantly differ between the two scenarios in the first CPR–intubation sequence. Outcomes of CPR quality metrics, intubation time, and intubation success rate in the second and third sequences did not differ between the two scenarios, except that the intubation time in the third sequence was significantly longer in the regurgitation scenario (16.5 vs. 9.0 s, *P* = 0.010) than in the clean airway scenario (Table 1). However, compared to the clean airway scenario, the trends of lower CCF, greater longest interruption duration, longer intubation time, and lower intubation success rate in the regurgitation scenario were consistent throughout the three sequences, but these did not achieve statistical significance. Post–hoc power analysis revealed the study power to be 95%, 76%, and 75% respective for detecting the significant difference of CCF, compression depth, and the longest interruption between the two groups.

**Table 1:** Primary and secondary outcomes by the type of airway during cardiopulmonary resuscitation

Outcomes	Oropharyngeal regurgitation	Clean airway	*P*
**The first CPR-intubation sequence**			
Number of EMT-P	54	54	
CCF (%), median (IQR)^a^	79.6 (73.9–85.0)	85.1 (81.8–90.0)	< 0.001
Compression depth (cm), median (IQR)^a^	5.2 (4.9–5.4)	5.4 (5.1–5.6)	< 0.001
Compression rate (bpm), median (IQR)^a^	102.0 (95.0–106.0)	102.0 (98.0–105.0)	0.295
The longest interruption (second), median (IQR)^a^	4.0 (3.0–6.0)	3.0 (2.0–4.0)	< 0.001
Intubation time (second), median (IQR)^a^	13.0 (8.0–16.0)	10.0 (7.0–14.0)	0.141
Successful intubation, n (%)^b^	19 (35.2)	43 (79.6)	< 0.001
Esophageal intubation, n	0	1	
**The second CPR-intubation sequence**			
Number of EMT-P	35	10	
CCF (%), median (IQR)^c^	80.0 (68.6–85.0)	84.5 (75.0–90.0)	0.104
Compression depth (cm), median (IQR)^c^	5.2 (4.9–5.4)	5.3 (5.0–5.5)	0.387
Compression rate (bpm), median (IQR)^c^	101.0 (95.3–105.0)	100.5 (95.0–104.0)	0.827
The longest interruption (second), median (IQR)^c^	4.0 (3.0–7.0)	4.0 (2.0–5.0)	0.240
Intubation time (second), median (IQR)^c^	12.0 (9.0–17.8)	11.5 (11.0–14.0)	0.784
Successful intubation, n (%)^d^	16 (45.7)	6 (60.0)	0.431
Esophageal intubation, n	1	1	
**The third CPR-intubation sequence**			
Number of EMT-P	18	3	
CCF (%), median (IQR) ^c^	77.3 (66.7–81.8)	84.2 (79.0–88.9)	0.159
Compression depth (cm), median (IQR)^c^	5.0 (4.8–5.5)	4.8 (4.8–5.0)	0.264
Compression rate (bpm), median (IQR)^c^	97.5 (94.0–104.0)	104.0 (97.3–107.8)	0.450
The longest interruption (second), median (IQR)^c^	6.0 (5.0–8.0)	3.0 (2.3–5.3)	0.093
Intubation time (second), median (IQR)^c^	16.5 (14.0–20.0)	9.0 (6.0–10.5)	0.010
Successful intubation, n (%)^d^	9 (50.0)	3 (100.0)	0.114
Esophageal intubation, n	2	0	
**Failure of all three CPR-intubation sequences**	7 (13.0)	0 (0)	0.006

Abbreviation: *EMT-P* emergency medical technician-paramedic, *CCF* chest compression fraction, *IQR* interquartile range, *bpm* beats per minute

^a^ Wilcoxon rank sum test

^b^ McNemar test

^c^ Mann–Whitney U test

^d^ Pearson's chi-squared test

As presented in Table 2, the longest interruption (*P* = 0.033) and intubation time (*P* = 0.005) in the oropharyngeal regurgitation scenario were significantly different among the three CPR–intubation sequences. However, in the clean airway scenario, the three CPR–intubation sequences had similar CPR quality metrics and intubation time.

**Table 2:** Comparison of cardiopulmonary resuscitation quality metrics and intubation time among the three cardiopulmonary resuscitation-intubation sequences by the type of airway during cardiopulmonary resuscitation

	Oropharyngeal regurgitation		Clean airway	
Variables	*P*	Post-hoc	*P*	Post-hoc
CCF	0.471	NA	0.848	NA
Compression depth	0.746	NA	0.059	NA
Compression rate	0.587	NA	0.722	NA
The longest interruption	0.033	1 < 3^a^	0.178	NA
Intubation time	0.005	1 < 3^b^, 2 < 3^c^	0.305	NA

Abbreviation: *CCF* chest compression fraction, *NA* not applicable, *1, 2, 3* the first, second, and third CPR-intubation sequence

^a^ The longest interruption in the first vs. third CPR-intubation sequence, *P* = 0.010

^b^ Intubation time in the first vs. third CPR-intubation sequence, *P* = 0.002

^c^ Intubation time in the second vs. third CPR-intubation sequence, *P* = 0.012

In the oropharyngeal regurgitation scenario, multivariate analysis revealed successful intubation to be significantly associated with compression depth (HR = 0.47, 95% CI, 0.24–0.91) but not with CCF, compression rate, or longest interruption duration. In the clean airway scenario, none of the CPR quality metrics were related to successful intubation (Table 3).

**Table 3:** Multivariate analysis for the association of successful intubation with cardiopulmonary resuscitation quality metrics and the number of intubation by the type of airway during cardiopulmonary resuscitation

	Oropharyngeal regurgitation			Clean airway		
Variables	HR	95% CI	*P*	HR	95% CI	*P*
CCF	0.99	0.95–1.03	0.603	1.03	0.96–1.10	0.407
Compression depth	0.47	0.24–0.91	0.026	1.08	0.40–2.89	0.886
Compression rate	1.02	0.98–1.05	0.331	1.02	0.96–1.07	0.565
The longest interruption	0.86	0.74–1.01	0.061	1.10	0.79–1.55	0.569
The number of intubation	1.10	0.73–1.65	0.662	0.98	0.51–1.87	0.954

Abbreviation: *CCF* chest compression fraction, *HR* hazard ratio, *CI* confidence interval

## Discussion

This manikin simulation study revealed regurgitation to be associated with a lower first-pass success rate and inferior CPR quality, including CCF, compression depth, and longest interruption duration, during the first CPR–intubation sequence. Similar trends of the impact of regurgitation on CPR quality and intubation were also observed during the second and third sequences, but it did not achieve statistical significance. In the oropharyngeal regurgitation scenario, the intubation time and longest interruption duration were significantly greater after failed intubation, and successful intubation was independently and negatively associated with compression depth. However, in the clean airway scenario, failed intubation did not affect the CPR quality or intubation time of the following CPR–intubation sequences, and none of the CPR quality metrics were associated with successful intubation.

High-quality CPR improves survival after cardiac arrest. In patients with OHCA, CCF is independently and positively associated with survival in patients with shockable rhythms [14, 15] and with the likelihood of return of spontaneous circulation in patients with nonshockable rhythms [16]. Deeper chest compressions are associated with superior survival and functional outcomes [17]. In the present study, although CPR quality was reduced by oropharyngeal regurgitation, all CPR quality metrics still met the recommendation of the American Heart Association, including a CCF of > 60%, compression rate of 100–120/min, compression depth of 5–6 cm, and compression pause duration of < 10 s [18]. These results may be due to the experience of the EMT-Ps, suggesting that proper training can help EMT-Ps maintain a certain level of CPR quality, even when dealing with oropharyngeal regurgitation.

Oropharyngeal regurgitation was associated with decreased survival to discharge in patients with cardiac-related OHCA [2]. Two strategies of airway management have been proposed to attenuate the negative effect of regurgitation on CPR quality. The first is the use of an SGA device. Studies have observed airway management with an SGA during CPR to be associated with higher CCF, shorter hands-off time, higher intubation success rates, and fewer required attempts than ETI [8–10, 19]. However, a cadaver study reported that ETI was associated with a lower regurgitation rate than was EasyTube insertion [6]. The AIRWAYS-2 Randomised Clinical Trial demonstrated that regurgitation and aspiration were significantly more common in the i-gel group than in the ETI group during or after advanced airway management [20]. Use of SGA devices might be associated with various complications in addition to aspiration, such as trauma and nerve injuries. Therefore, proper device use and awareness of the patients with high risks of complications are vital [21].

Another strategy of airway management for regurgitation during CPR is mitigating the regurgitated gastric contents during intubation using passive drainage or active drainage. Two methods of passive drainage have been reported: semiprone positioning and intentional oesophageal intubation. Fevang et al. described the successful intubation and resuscitation of two children who experienced cardiac arrest and water submersion with massive regurgitation; a semiprone position was employed for passive drainage of the regurgitated contents without interruption of chest compression, and the children were discharged from hospital without any sequelae [22]. Sorour et al.[23] and Kornhall et al.[24] reported initially inserting the endotracheal tube into the oesophagus and inflating the cuff balloon to stop the gastric contents from being regurgitated into the mouth. The gastric contents were drained passively through the endotracheal tube in the oesophagus. After brief suctioning, the laryngeal view was clear, enabling successful intubation.

Suction-assisted laryngoscopy and airway decontamination (SALAD) technique, developed by DuCanto et al., is a method of active drainage of airway contaminants during intubation [25, 26]. The main steps of the SALAD technique are the hypopharyngeal withdrawal and reinsertion of a large-bore rigid suction catheter to the left of the laryngoscope for active and continued drainage of blood or regurgitated gastric contents after initial suctioning the mouth. Studies have demonstrated that SALAD training can help paramedics perform better intubation of obstructed airways, including significantly shorter intubation times and a higher first-pass success rate [27, 28]. Because maintaining the semiprone position for chest compression in adults with OHCA is difficult, intentional oesophageal intubation and the SALAD technique in the supine position may be preferable for mitigating regurgitation and facilitating intubation during CPR. The effect of these techniques on CPR quality in the presence of oropharyngeal regurgitation deserves further research.

Our study has several limitations. First, the EMP-Ts participating in this study performed CPR and intubation on a modified airway-CPR manikin instead of on real patients. Because the manikin cannot realistically mimic the feel of the human chest during CPR or the human airway during airway management, our results need to be verified in the real world. Second, we used a chest compression plate with a compression pad squeezing a pump to simulate regurgitation in the airway, but the exact volume of regurgitated material was unknown. Thus, we could not analyse the relationship of compression depth or compression rate with regurgitation volume and could not better comprehend the differences between our modified airway-CPR manikin and real patients with regurgitation during CPR. However, our modified airway-CPR manikin is the first model simulating regurgitation during CPR and can be further modified by adjusting the diameter of the tube between the oesophagus and outflow port to change the amount of regurgitation with each compression. Finally, because we wanted to know the CPR quality during second and third CPR–intubation sequences after failed intubation attempts, we did not restrict the volume of regurgitated gastric content in the subsequent sequences. The insignificant results during second and third CPR–intubation sequences might come from the small sample size. However, in the real world, the gastric contents may be cleared from the stomach after an initial failed intubation attempt, and the subsequent intubation attempts and CPR may not be influenced by regurgitation.

In conclusion, this manikin simulation study demonstrated a considerable difference in CPR quality between ETI with and without oropharyngeal regurgitation, especially during the first CPR–intubation sequence. Oropharyngeal regurgitation during CPR with ETI was significantly associated with the following differences in CPR quality metrics: lower CCF and compression depth and greater longest interruption duration. Successful intubation in the case of oropharyngeal regurgitation was independently and negatively associated with chest compression depth during CPR. Further investigations are warranted to validate our results. Our findings imply that advanced training might be necessary to improve EMT-P competency in performing CPR with ETI during oropharyngeal regurgitation.

## Supplementary Information

**Additional file 1: Supplementary Fig. 1** Study protocol. All EMT-Ps were assigned to participate in either the oropharyngeal regurgitation or clean airway scenario and switched to the other scenario after they had finished their first simulation. In each scenario, each EMT-P took turns playing three roles: airway manager, first compressor, and second compressor. For each simulation, the airway manager performed BVM ventilation and intubation, and the first and second compressors alternately provided chest compressions for every five cycles of CPR, with a compression-to-ventilation ratio of 30:2. Abbreviations: EMT-Ps, emergency medical technician-paramedics.

**Additional file 2: Supplementary Fig. 2** Definition of start and end of CPR–intubation sequence. **A** The airway manager was asked to perform intubation after the first five cycles of CPR. Therefore, the first CPR–intubation sequence started at the end of the last chest compression of the fifth cycle of CPR. If the first intubation attempt ended with successful intubation during the compression segment, this compression segment was a part of the first CPR–intubation sequence. **B** If the first intubation attempt failed during the compression segment and no reintubation was attempted in the same segment, this compression segment was a part of the first CPR–intubation sequence, and the following hands-off segment of manual ventilation was a part of the second CPR–intubation sequence. **C** If the first intubation attempt failed during the compression segment and reintubation was attempted in the same segment, this compression segment was divided into two parts at the end of the first CPR-intubation sequence. The first was a part of the first CPR–intubation sequence, and the second was a part of the second sequence. **D** If the first intubation attempt ended during the hands-off segment and followed by two manual ventilations, this hands-off segment was divided into two parts—the first part in the first sequence, and the second part of ventilations in the second sequence. The second and third CPR–intubation sequences were defined using the same rules. Abbreviations: CPR, cardiopulmonary resuscitation; C, chest compression segment; H, hands-off segment.

**Additional file 3: Supplementary Video 1.** The simulated oropharyngeal regurgitation during chest compression.

## Data Availability

The data that support the findings of this study are available from the corresponding author upon reasonable request.

## Abbreviations

CPR: Cardiopulmonary resuscitation
CCF: Chest compression fraction
OHCA: Out-of-hospital cardiac arrest
BVM: Bag valve mask
ETI: Endotracheal intubation
SGA: Supraglottic airway
