# Supplementary figures and images for: Compromised cardiopulmonary resuscitation quality due to regurgitation during endotracheal intubation: a randomised crossover manikin simulation study
Source: BMC Emerg Med. 2022 Jul 9;22:124. doi: 10.1186/s12873-022-00662-0 (PMC9270833; doi:10.1186/s12873-022-00662-0)

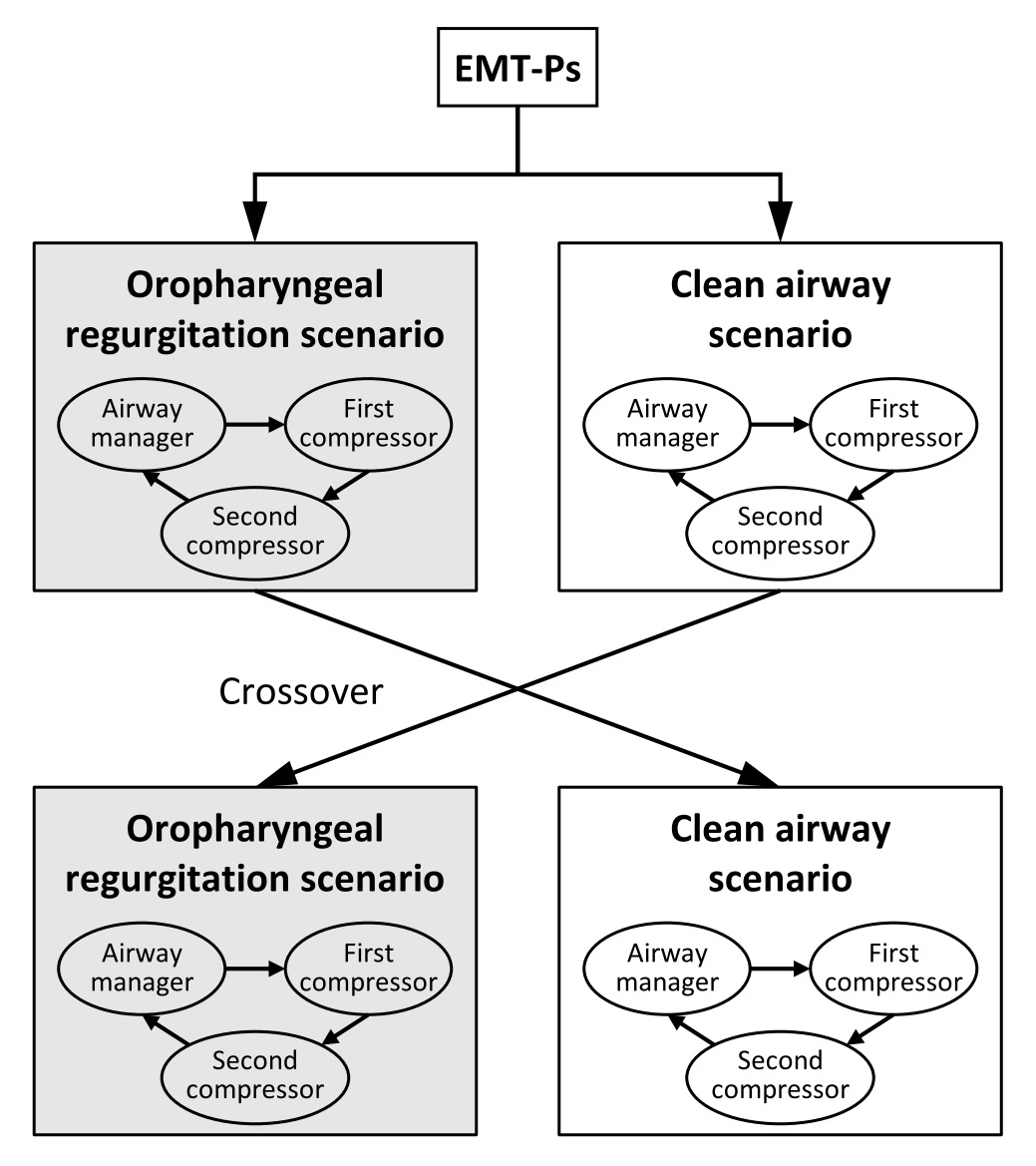

Supplement: Supplementary file 1 — Additional file 1: Supplementary Fig. 1 Study protocol. All EMT-Ps were assigned to participate in either the oropharyngeal regurgitation or clean airway scenario and switched to the other scenario after they had finished their first simulation. In each scenario, each EMT-P took turns playing three roles: airway manager, first compressor, and second compressor. For each simulation, the airway manager performed BVM ventilation and intubation, and the first and second compressors alternately provided chest compressions for every five cycles of CPR, with a compression-to-ventilation ratio of 30:2. Abbreviations: EMT-Ps, emergency medical technician-paramedics. [file 12873_2022_662_MOESM1_ESM.jpg]

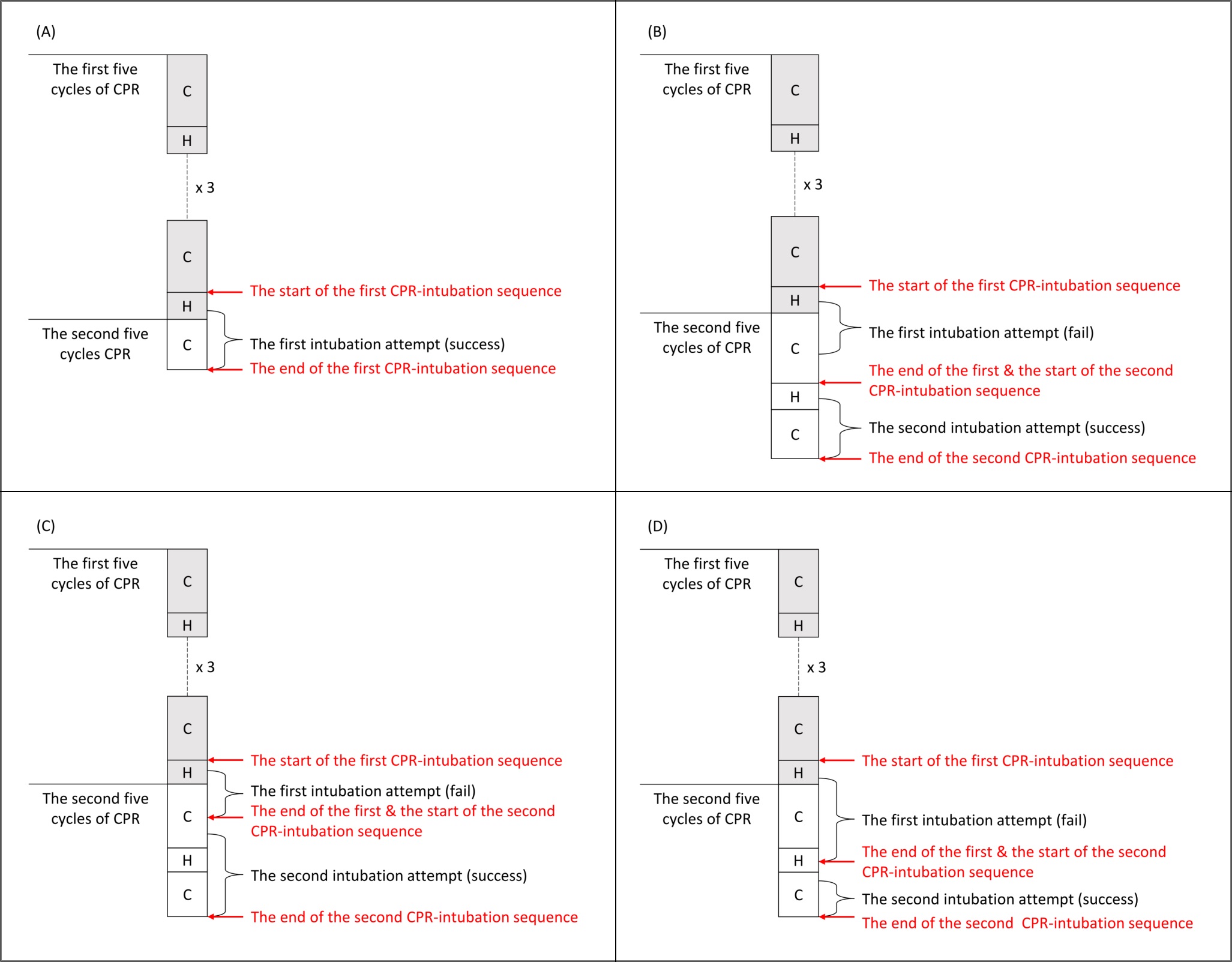

Supplement: Supplementary file 2 — Additional file 2: Supplementary Fig. 2 Definition of start and end of CPR–intubation sequence. A The airway manager was asked to perform intubation after the first five cycles of CPR. Therefore, the first CPR–intubation sequence started at the end of the last chest compression of the fifth cycle of CPR. If the first intubation attempt ended with successful intubation during the compression segment, this compression segment was a part of the first CPR–intubation sequence. B If the first intubation attempt failed during the compression segment and no reintubation was attempted in the same segment, this compression segment was a part of the first CPR–intubation sequence, and the following hands-off segment of manual ventilation was a part of the second CPR–intubation sequence. C If the first intubation attempt failed during the compression segment and reintubation was attempted in the same segment, this compression segment was divided into two parts at the end of the first CPR-intubation sequence. The first was a part of the first CPR–intubation sequence, and the second was a part of the second sequence. D If the first intubation attempt ended during the hands-off segment and followed by two manual ventilations, this hands-off segment was divided into two parts—the first part in the first sequence, and the second part of ventilations in the second sequence. The second and third CPR–intubation sequences were defined using the same rules. Abbreviations: CPR, cardiopulmonary resuscitation; C, chest compression segment; H, hands-off segment. [file 12873_2022_662_MOESM2_ESM.jpg]
